# Detection of circulating *Mycobacterium tuberculosis*-specific DNA by droplet digital PCR for vaccine evaluation in challenged monkeys and TB diagnosis

**DOI:** 10.1038/s41426-018-0076-3

**Published:** 2018-04-24

**Authors:** Neng Song, Yang Tan, Lingyun Zhang, Wei Luo, Qing Guan, Ming-zhe Yan, Ruiqi Zuo, Weixiang Liu, Feng-ling Luo, Xiao-Lian Zhang

**Affiliations:** 1 State Key Laboratory of Virology and Medical Research Institute, Hubei Province Key Laboratory of Allergy and Immunology and Department of Immunology, Wuhan University School of Basic Medical Sciences, Wuhan, 430071 China; 2 Wuhan Medical Treatment Center, Wuhan, 430071 China

## Abstract

*Mycobacterium tuberculosis* (*M. tb*) is emerging as a more serious pathogen due to the increased multidrug-resistant TB and co-infection of human immunodeficiency virus (HIV). The development of an effective and sensitive detection method is urgently needed for bacterial load evaluation in vaccine development, early TB diagnosis, and TB treatment. Droplet digital polymerase chain reaction (ddPCR) is a newly developed sensitive PCR method for the absolute quantification of nucleic acid concentrations. Here, we used ddPCR to quantify the circulating virulent *M. tb*-specific CFP10 (10-kDa culture filtrate protein, Rv3874) and Rv1768 DNA copy numbers in the blood samples from Bacille Calmette-Guerin (BCG)-vaccinated and/or virulent *M. tb* H37Rv-challenged rhesus monkeys. We found that ddPCR was more sensitive compared to real-time fluorescence quantitative PCR (qPCR), as the detection limits of CFP10 were 1.2 copies/μl for ddPCR, but 15.8 copies/μl for qPCR. We demonstrated that ddPCR could detect CFP10 and Rv1768 DNA after 3 weeks of infection and at least two weeks earlier than qPCR in *M*.*tb* H37Rv-challenged rhesus monkey models. DdPCR could also successfully quantify CFP10 and Rv1768 DNA copy numbers in clinical TB patients’ blood samples (active pulmonary TB, extrapulmonary TB (EPTB), and infant TB). To our knowledge, this study is the first to demonstrate that ddPCR is an effective and sensitive method of measuring the circulating CFP10 and Rv1768 DNA for vaccine development, bacterial load evaluation in vivo, and early TB (including EPTB and infant TB) diagnosis as well.

## Introduction

Tuberculosis (TB) is an emerging health problem with high morbidity and mortality, particularly in developing countries. *Mycobacterium tuberculosis* (*M. tb*) is the causative agent of TB. Approximately, one-third of the world’s population has been infected with *M. tb*, and 10% of these individuals show clinical symptoms. Individuals with compromised immune systems such as human immunodeficiency virus (HIV) patients, malnutrition diabetes patients, or tobacco users have a much higher risk of *M. tb* infection^1^. The only available TB vaccine is the attenuated strain of *Mycobacterium bovis*, termed as Bacillus Calmette-Guerin (BCG), which has limited efficacy, partly due to the absence of immunogenic proteins. Therefore, the development and evaluation of an efficacious TB vaccine remains to be a major challenge to the TB-vaccine designers.

*M. tb* typically affects the lungs, inducing pulmonary TB (PTB), and also affects other organs and tissues including intestines, bones, joints, kidneys, brain, and spine leading to extrapulmonary TB (EPTB). Following infection, *M. tb* can also persist in the host without any clinical symptoms, resulting in latent TB infection (LTBI). This long-term stable infection status can evolve into active TB when the host immunity is low, and many TB cases reflect reactivation in LTBI individuals migrating or traveling across borders between countries and disease-burdened regions^2^. Children in close contact with active TB patients have an increased risk of infection. Among children, the greatest numbers of TB cases are observed in children less than 5-years-old, referred to as infant TB. Early TB diagnosis and treatment is important for patient outcomes and spread prevention. Therefore, a rapid and sensitive detection method for *M. tb* diagnosis and vaccine development is urgently needed^3^.

In many developing countries, TB diagnosis is based on clinical findings, chest radiography, and acid-fast bacilli (AFB) detection in smears and sputum cultures, but these methods do not provide sufficiently rapid results for clinicians^4^. Tuberculin skin test (TST) and interferon-gamma release assay (IRGA) are also used for active TB and LTBI diagnosis. However, the TST with purified protein derivative (PPD) has a poor specificity because BCG vaccination and exposure to nontuberculosis mycobacteria (NTM) produce similar responses, as genuine *M. tb* infection. Particularly, the diagnosis of EPTB and infant TB is extremely difficult due to insufficient specimen material and the scarcity of bacilli in specimens^4^. Therefore, it is necessary to develop a novel sensitive method that can specifically identify virulent *M. tb* for active pulmonary TB, EPTB, and infant TB.

In recent years, polymerase chain reaction (PCR) and other methods for amplifying DNA and RNA have been developed, which enable TB diagnosis in several hours^5–7^. For example, Xpert MTB/RIF (Cepheid, Sunnyvale, CA, USA), a rapid automatic system based on real-time PCR and molecular beacon technology, used to detect *M. tb* genes from untreated sputum samples in less than 2 h^8,9^.The real-time fluorescence quantitative PCR (qPCR) was used to quantify BCG DNA in skin biopsy specimens of the BCG-vaccinated adults^10^. Digital polymerase chain reaction (dPCR) is a PCR method for the absolute quantification of nucleic acid concentrations. More recently, a new method, referred to as droplet digital PCR (ddPCR), has gained attention as a more precise and less-subjective assay to quantify DNA amplification^11,12^. However, ddPCR has not been reported to detect virulent *M. tb*-specific DNA copy numbers in the blood samples, particularly for infant TB, and has not been used to detect bacterial loads at different time courses in *M. tb-* infected animal models and evaluate the vaccine protective efficacy in vivo.

In the present study, we collected the blood samples from Chinese TB patients (active PTB, EPTB, and infant TB), healthy donors (HDs), and virulent *M. tb* H37Rv-infected rhesus monkeys at different time courses, and subsequently used the ddPCR method to quantify virulent *M. tb*-specific CFP10 (10-kDa culture filtrate protein, Rv3874) and Rv1768 copy numbers for evaluating the vaccine protective efficacy in vivo and TB diagnosis.

## Results

### Determination of optimum concentration for the PCR probe and the standard curves of qPCR and ddPCR

**Fig. 1:**
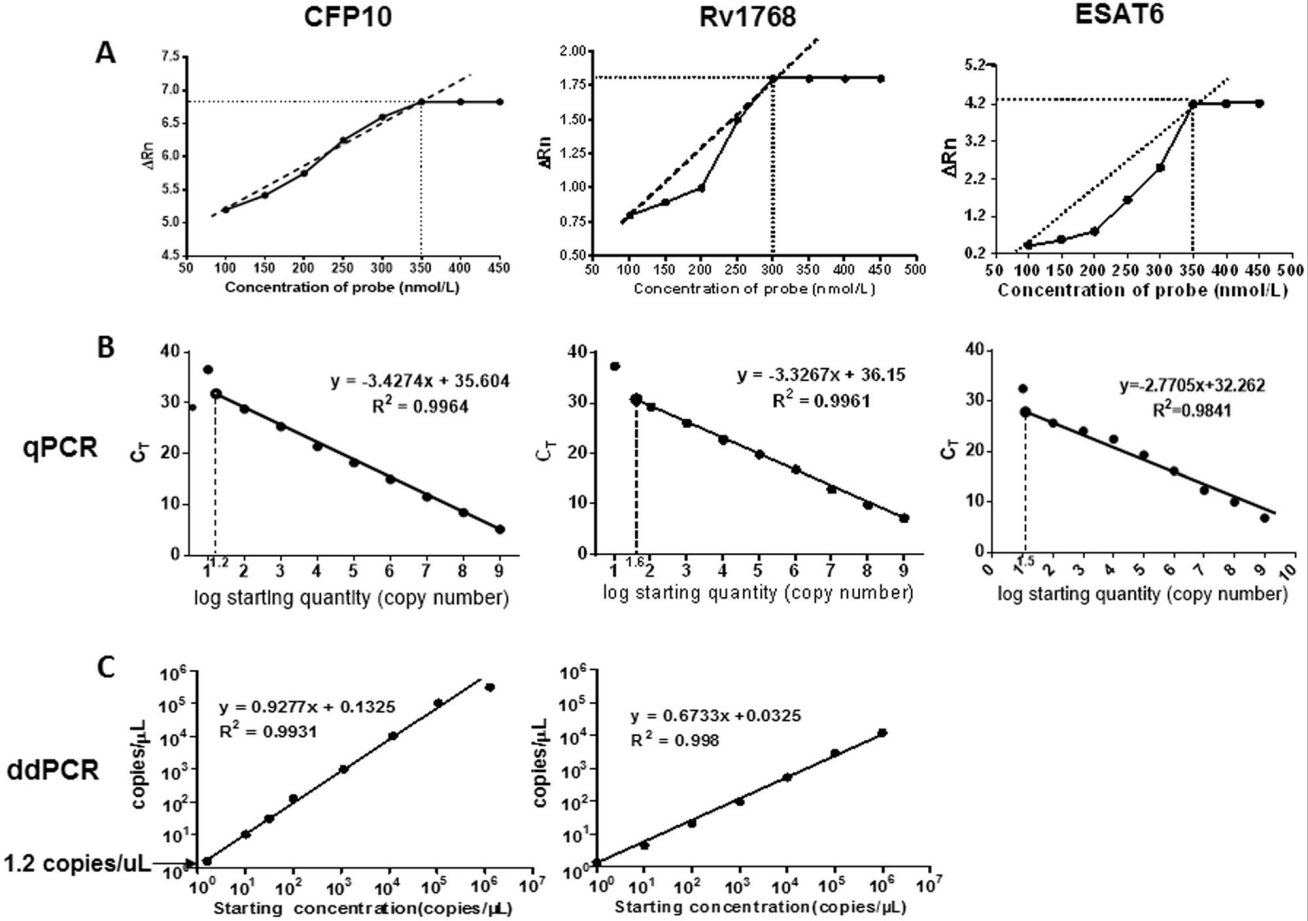
Standard curves and detection range of recombinant plasmids using qPCR and ddPCR. **a** Optimum concentrations of CFP-10, Rv1768, and ESAT6 probes. Each sample was analyzed in three repetitive wells in one reaction, and each experiment was repeated three times. **b** Using qPCR, the CFP10 linear regression formula was *y* = −3.4274x + 35.604 (*r*^2^ = 0.9964), the Rv1768 linear regression formula was *y* = −3.3267x + 36.15 (*r*^2^ = 0.9961), and the ESAT-6 linear-regression formula was *y* = −2.7705x + 32.262 between the plasmid copy numbers and Ct values. **c** The linearity range of ddPCR for quantifying pET28a-CFP10 and pET28a-Rv1768 plasmid DNA. The formulae were *y* = 0.9277x + 0.1352 (*r*^2^ = 0.9931) for CFP10 and *y* = 0.6733x + 0.0325 (*r*^2^ = 0.998) for Rv1768.

Different probe concentrations were set as 100, 150, 200, 150, 200, 350, 400, and 450 nmol/L. The fluorescence signal intensity demonstrated that 350 nmol/L concentration was optimal for CFP10 and ESAT6, and 300 nmol/L concentration for Rv1768, by qPCR, respectively. Thus, the probe concentration in the PCR reaction was selected as 350 nmol/L for CFP10 and ESAT6, 300 nmol/L for Rv1768 (Fig. 1a).

The recombinant plasmid DNA were 10-fold serial diluted as 10^9^, 10^8^, 10^7^, 10^6^, 10^5^, 10^4^, 10^3^, 10^2^, and 10^1^ copies/μl, and subsequently 2 μL of each diluted plasmid was used for the PCR reaction. In qPCR, the plasmids were further diluted as 10^1.2^, 10^1.4^, 10^1.6^, and 10^1.8^copies/μl to acquire the accurate detection limits.

For qPCR, the plasmid copy numbers were determined and were set as the X-axis using the following formula, the copy numbers of *M. tb*-specific genes (concentration in ng × 6.022 × 10^23^)/(genome length × 10^9^ × 650)^13^. The corresponding Ct value was set as the y-axis [Ct value = *m* (log quantity) + *b*], in which *m* and *b* represent the slope and intercept, respectively. As shown in Fig. 1b, the standard curve was deviated when the recombinant plasmid concentration was below 15.8 copies/μl (CFP10) or 39.8 copies/μl (Rv1768), and a linear correlation (*r*^2^ = 0.9964 and *r*^2^ = 0.9961) was observed between the copy number and the Ct value. The corresponding linear regression formula was *y* = −3.4274 × + 35.604 for CFP10, and *y* = −3.3267 × + 36.15 for Rv1768.

We have also compared the linear relations of different *M. tb*-specific genes (CFP10, Rv1768, and ESAT6) by qPCR. We found that the linear correlations of CFP10 and Rv1768 were better than ESAT-6 (*R*^2^ = 0.9964 for CFP10, *R*^2^ = 0.9961 for Rv1768, *R*^2^ = 0.9841 for ESAT6). So we used CFP10 and Rv1768 genes in the following experiments.

For ddPCR, we also observed a strong linear correlation (*R*^2^ = 0.9931, *R*^2^ = 0.9980) with the following corresponding formula: *y* = 0.9277x + 0.1325 for CFP10, and *y* = −0.6733x + 0.1325 for Rv1768 (Fig. 1c). For CFP10, the standard curve was normal, even when the recombinant plasmid concentration was as low as 1.2 copies/μl; thus, the detection sensitivity was 15.8 copies and 1.2 copies for qPCR and ddPCR, respectively.

### qPCR and ddPCR analysis of bacterial loads in BCG vaccination and *M. tb*-challenged rhesus monkeys

**Fig. 2:**
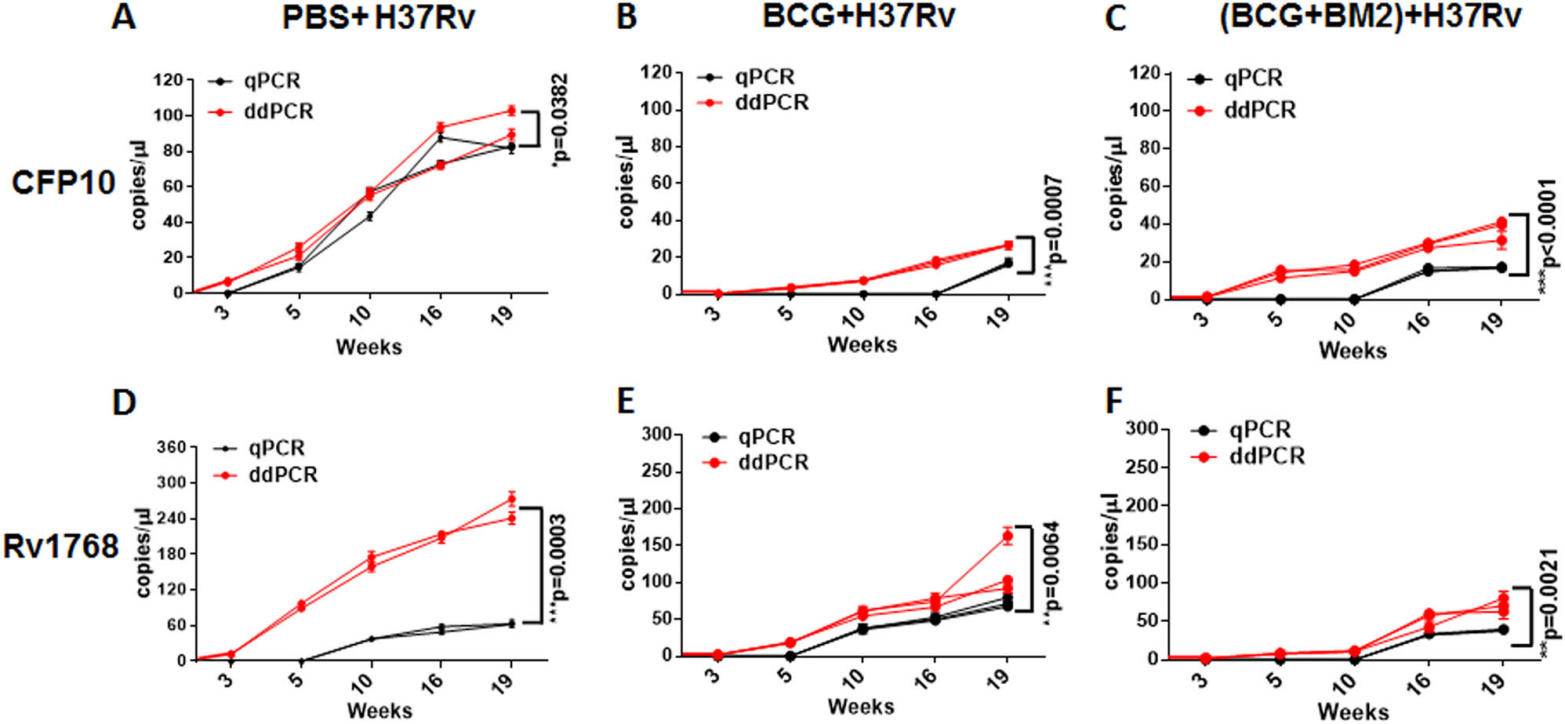
Comparison of qPCR and ddPCR analysis of CFP10 and Rv1768 gene copy numbers in whole-blood DNA of the BCG-immunized and *M. tb*-infected RM models. Each sample measured three times and each point represents the mean result of replicates. Each line (red or black) represents one monkey. ANOVA was used to compare the experimental data from qPCR and ddPCR. Differences were considered statistically significant for *p* < 0.05. a-c: for CFP10; d-f: for Rv1768. a-f have been provided in the figure legend.

To test the performance of ddPCR as a tool to evaluate the protective efficacy in vaccine studies performed in nonhuman primates, we used banked blood specimens from a previously reported monkey study^14^. Because of the expense, a total of eight rhesus monkeys (RM) were used in three groups (PBS (*n* = 2), BCG (*n* = 3), and BCG + BM2 (ssDNA aptamer “antibody” BM2 specifically bound to the mannose-capped lipoarabinomannan (ManLAM) of BCG) (*n* = 3) groups)^14^. Briefly, at week -5, the RMs were vaccinated with BCG or BCG + BM2. At week 0, all RMs were infected with 100 CFUs of *M. tb* H37Rv. Then blood samples were collected and stored at 3, 5, 10, 16, 19 weeks after H37Rv infection^14^. The bacterial CFU burden in the lung has been detected and histopathological analysis has been performed to confirm that BCG/BCG + BM2 vaccine groups elicited protective efficiency, compared with the PBS group in our previous report^14^. In the present study, we further detected the CFP-10 and Rv1768 genes for parallel comparison of each banked blood samples using ddPCR and qPCR, respectively (Fig. 2).

**Fig. 3:**
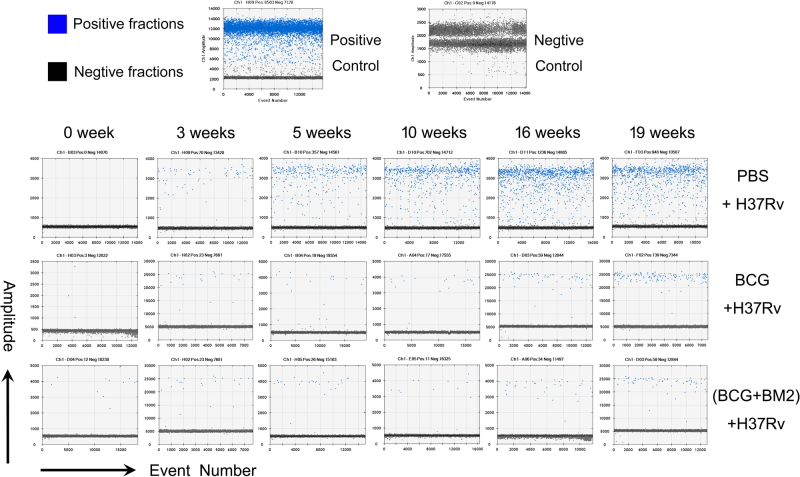
**Representative data of the original microdrop pictures of the CFP10 ddPCR results for** ***M. tb*** **-infected RMs**

Using ddPCR, the original microdrop pictures of CFP10 are shown in Fig. 3. The CFP10 and Rv1768 gene copy numbers were detected after 3 weeks of infection and at least 2 weeks earlier using ddPCR, compared to qPCR (Fig. 2a, d), which further demonstrated that ddPCR was more sensitive than qPCR. ANOVA was used to compare the experimental data obtained from qPCR and ddPCR. A significant difference was observed between the two PCR methods, as shown in Fig. 2a, d, *p* values = 0.0302 and 0.0003, respectively.

Moreover, we observed much lower *M. tb* gene copy numbers in the BCG group, compared to the PBS group (Fig. 2a, b, d, e and Fig. 3). The CFP10 DNA copy numbers were 34.3–41.1 copies/μl in the BCG-vaccinated group and 93.4–104 copies/μl in the PBS-treated group (Fig. 2a, b), respectively, while the mean Rv1768 DNA copy numbers were 96.3–170.0 copies/μl in the BCG-vaccinated group and 245.0–282.0 copies/μl in the PBS-treated group at week 19, post the infection, by ddPCR (Fig. 2d, e), suggesting that the BCG-vaccinated group had much less bacterial load and a better protective effect than the PBS-treated group. We further observed much fewer CFP10 gene copy numbers (19–20.8 copies/μl) in the BCG vaccinated + BM2 treatment group than in the BCG-vaccinated group (34.3–41.1 copies/μl) (Fig. 2b,c, and Fig. 3). Similarly, fewer lower Rv1768 gene copy numbers (69.0–86.0 copies/μl) were detected in the BCG vaccinated + BM2 treatment group than in the BCG-vaccinated group (96.3–170.0 copies/μl) (Fig. 2e, f). These data suggest that BCG vaccination plus BM2 adjuvant has a stronger protective effect than BCG vaccination alone (Fig. 2). Other data (bacterial burden, radiologic, pathology, etc) have been provided to confirm the BCG/BCG + BM2 groups elicited protection in our previous study^14^. Our present data strongly showed that ddPCR had much higher sensitivity than qPCR for bacterial load evaluation and vaccine development.

### DdPCR and qPCR analysis of the blood samples from patients and HDs

We also measured the CFP10 and Rv1768 gene copy numbers in the blood samples of Chinese TB patients and HDs, using both ddPCR and qPCR for parallel comparison for each blood sample. The clinical characteristics of all participants are shown in Table 1. In total, 26 TB patients samples including 10 PTB adult patients samples, 10 EPTB adult patients samples, 6 PTB infant patients samples, and 16 HD samples including 10 adult HDs samples, and 6 infant HDs samples were used for both ddPCR and qPCR analysis.

**Table 1:** Clinical characteristics of study participants

Characteristic	PTB (*n* = 10)	EPTB (*n* = 10)	HDs (*n* = 10)	Infant PTB (*n* = 6)	Infant HDs (*n* = 6)
Age (*y*)	30.15 ± 12.15	33.23 ± 10.79	32.45 ± 8.95	0.84 ± 0.46	1.06 ± 0.37
Female gender	5/10 (50.00%)	5/10 (50.00%)	5/10 (50.00%)	3/6 (50.00%)	3/6 (50.00%)
Clinical presentations					
TB symptoms	10/10 (100.00%)	10/10 (100.00%)	0/10	6/6 (100.00%)	0/6
Abnormal chest X-ray findings	9/10 (90.00%)	3/10 (30.00%)	0/10	4/6 (66.67%)	0/6
Other body parts X-ray or CT/MRI findings	n.a.	10/10 (100%)	n.a.	n.a.	n.a.
Laboratory findings					
White blood cell count (cells/µl)	7.09 (4.83–8.05)	5.83 (4.35–6.75)	5.89 (5.51–7.50)	11.35 (11.23–12.89)	11.85 (11.10–12.57)
Lymphocyte count (cells/µl)	1.54 (0.86–1.62)	1.47 (0.99–1.57)	2.23 (1.92–2.56)	0.62 (0.48–0.67)	0.58 (0.55–0.63)
Positive sputum acid-fast staining	3/10 (30.00%)	1/10 (10.00%)	n.a.	2/6 (33.33%)	n.a.
Sputum culture	9/10 (90.00%)	2/10 (20.00%)	n.a.	4/6 (66.67%)	n.a.
Positive IGRA^c^	7/10 (70.00%)	8/10 (80.00%)	n.a.	3/6 (50.00%)	n.a.
ELISPOT	n.a.	n.a.	0/10	n.a.	0/6

Data are presented as no. of positive/no. of tested (%), mean ± standard deviation or median (inter-quantile range)

*n.a.* not available; *PTB* pulmonary tuberculosis; *EPTB* extra pulmonary tuberculosis; *IGRA* IFN-γ release assay (commercial IFN-γ T-SPOT TB assays were used); *ELISPOT* commercial IFN-γ release enzyme-linked immunospot assay

As shown in Table 2, we detected CFP10 or Rv1768 DNA and obtained positive results for all patients using ddPCR. The sensitivity, specificity, and detectable rate were all 100% by using ddPCR. However, the sensitivity, specificity, and detectable rate were 65% (95% CI: 44%–83%), 100%, and 65%, respectively, by using qPCR. Among 26 TB patients, we observed that CFP10 DNA was not detectable (negative results) in 3 PTB adult patients, 2 EPTB adult patients, and 3 PTB infant patients by using qPCR. Similarly, Rv1768 DNA was not detectable in 3 PTB infant patients, as well, using qPCR (Table 2). For sputum acid-fast staining and sputum culture, the sensitivities were only 23% (95% CI: 8.97%–43.65%) and 58% (95% CI: 36.92%–76.65%), respectively. The above data strongly suggest that ddPCR has higher sensitivity than qPCR, sputum acid-fast staining, and sputum culture for TB diagnosis.

**Table 2:** Comparison between ddPCR and qPCR for TB diagnosis in the clinical blood samples

Blood samples	ddPCR				Total (no)	qPCR				Total (no)			Total (no)			Total (no)
	CFP10		Rv1768			CFP10		Rv1768			Sputum acid-fast staining			Sputum culture		
	+	−	+	−		+	−	+	−		+	−		+	−	
PTB patients	10	0	ND	ND	10	6	4	ND	ND	10	3	7	10	9	1	10
EPTB patients	10	0	ND	ND	10	8	2	ND	ND	10	1	9	10	2	8	10
PTB infant patients	6	0	6	0	6^a^	3	3	3	3	6^a^	2	4	6	4	2	6
Adult HDs	0	10	ND	ND	10	0	10	ND	ND	10	0	10	10	0	10	10
Infant HDs	0	6	0	6	6^a^	0	6	0	6	6^a^	0	6	6	0	6	6
Total	26	16	6^a^	6^a^	42	17	25	3^a^	9^a^	42	6	36	42	15	27	42

^a^Same blood samples from PTB infant patients and infant HDs were used for both Rv1768 and CFP10

*ND* not detected in this study, + positive, − negative

## Discussion

Through comparative genomic hybridization between virulent *M. tb* H37Rv and attenuated *M. bovis* BCG, BCG lost 129 open-reading frames in the course of evolution, and these regions are referred to as regions of deletion (RD). The Rv3874 (CFP10) gene belongs to RD1, and the Rv1768 gene belongs to RD14. CFP10 and ESAT-6 (Rv3875) are the two most predominant secretory proteins evaluated as candidate vaccine(s) and antigens in TB vaccine development and diagnosis^15,16^. IGRA, based on CFP10/ESAT-6, has been widely used in clinical diagnosis. Rv1768 is a proline-glutamic acid (PE) family member, which is also important for *M. tb* pathogenesis^17^.

DdPCR is a new generation-accurate-quantitative technique that is widely used for the detection of pathogenic microorganisms and rare mutations of molecular markers^18–22^. Unlike qPCR, analysis of the ddPCR results does not depend on a standard curve, which is easily affected by various factors^23^. DdPCR has considerably higher sensitivity and more multivariate analysis ability, and this method has been developed for measuring low copy numbers of hepatitis B virus (HBV), enterovirus, and several other pathogenic microorganisms^18–22^.

To our knowledge, the present study was the first to demonstrate that ddPCR could be used to detect changes in CFP10 and Rv1768 DNA copy numbers in an *M. tb* H37Rv-challenged RM model. The data revealed that ddPCR could detect *M. tb-*specific CFP10 and Rv1768 DNA at 3 weeks post *M. tb* infection in vivo, at least 2 weeks earlier than qPCR (Fig. 2). This result also indicated that *M. tb* might appear in the blood 3 weeks after *M. tb* infection in humans. Furthermore, ddPCR could be used not only for measuring bacterial loads, but also for vaccine evaluation. BCG is the only vaccine that has been widely used for the detection of TB, and the present study is the first to demonstrate that the protective efficiency of BCG plus adjuvant BM2 was much better than that of BCG alone, since bacterial loads were much less in the BCG plus adjuvant BM2 group than in the BCG group, after challenging with virulent *M. tb* H37Rv in RMs.

This study is also the first to report the quantification of virulent *M. tb* CFP10 and Rv1768 DNA copy numbers in TB patients’ whole-blood samples using ddPCR. Although some studies have reported the detection of *M. tb* conserved genes and *M. tb* complex copy numbers using ddPCR ^24,25^, Yang et.al detected IS6110 in the whole-blood samples from pulmonary TB and extrapulmonary TB patients^26^. The present study is the first to use ddPCR to diagnose infant TB patients. Due to the high cost of ddPCR, in total, only 26 TB patients (10 PTB, 10 EPTB and 6 infant PTB) and 16 HDs (10 adults and 6 infant HDs) samples were used in this study. Notably, application of ddPCR in clinical decision-making requires further studies involving larger numbers of patients and more comparisons such as blood culture, and combinations of other NAAT target genes, such as IS6110 ^27^. We will also test the same samples using ddPCR, following chemotherapy in our future study.

The peripheral-blood-based ddPCR could be developed into a minimally invasive, sensitive, and timely assistant diagnostic approach. These data demonstrate the potential of ddPCR to contribute to the early diagnosis of EPTB patients lacking respiratory symptoms and infant TB patients, from whom it is impossible to obtain efficient sputum samples. In the present study, we measured the CFP10 or Rv1768 gene copy numbers in active PTB patients, infant TB patients, EPTB patients, and HDs. Our results showed that the CFP10 or Rv1768 genes were not detectable in 11 TB patients using qPCR, but could successfully be detected in all patients (including EPTB patients and infant TB patients) using ddPCR (Table 2).

Our ddPCR method remarkably improves the detection sensitivity, compared to qPCR. The positive results by ddPCR and qPCR indicate previous infection or recent infection by the bacterium. Whether positive result representing dead or alive *M. tb* is needed for further verification by other methods, such as a chromosomal equivalence assay^28^.

In conclusion, compared to qPCR, ddPCR has advantages of being absolutely quantitative and having higher sensitivity. DdPCR has a great application prospect to assess bacterial loads for vaccination in vivo, and in timely TB diagnosis, as well.

## Materials and methods

### Ethics statement

The ethics committee of the Wuhan University School of Medicine, Wuhan Medical Treatment Center and Wuhan Children’s Hospital approved the present study. Written informed consent was obtained from all participants.

### Clinical specimens

Peripheral whole-blood samples from 26 TB patients including 16 active pulmonary tuberculosis (PTB) patients and sputum smear-positive or culture-positive including 10 adults and 6 infants, and 10 EPTB patients (biopsy or culture positive), were collected from Wuhan Medical Treatment Center, Shenzhen Third People’s Hospital, and all the patients received standard anti-TB therapy treatment for less than 3 weeks prior to the blood sample collection. All patients had a final clinical diagnosis of TB, which ruled out NTM as the causative agent in different samples, and all cases had typical symptoms. Patients receiving immunosuppressive regimens or infected with HIV were excluded. The whole-blood samples from 16 HDs (including 10 adults and 6 infants) were collected from Zhongnan Hospital of Wuhan University and Wuhan Children’s Hospital. All HDs had no TB, based on clinical examination and laboratory tests (such as TB-specific IFN-γELISPOT analysis). A 2-ml whole-blood sample was obtained from each patient or healthy donor and maintained in vacutainer tubes containing EDTA-2K. All blood samples were transported at 4 °C and processed within 6 h after collection.

### DNA extraction from whole blood of patients and healthy donors

Total DNA was extracted using a blood DNA extraction kit (Axygen, New York, USA), according to the manufacturer’s instructions. The principle of this kit was based on the efficient release of total DNA from anti-coagulated whole blood by proteinase K digestion, cell lysis, and heme/protein precipitation. A total of 200μl of whole blood was used for each extraction, and the purified DNA was eluted in a low-salt Tris buffer containing 0.5 mM EDTA, which enhanced the DNA solubility and protected the high-molecular weight DNA against nuclease degradation. DNA concentration was determined using the NanoDrop 2000 Ultramicro spectrophotometer (Thermo Scientific, Waltham, MA, USA), and different DNA samples were diluted to the same concentration, as the template for application. The DNA samples were stored at –80 °C until further use (<4 weeks).

### BCG vaccinations and *M. tb* H37Rv challenge in RM

The Institutional Animal Care and Use Committee of Wuhan University approved the animal experimental protocol. The virulent *M. tb* strain H37Rv (ATCC 93009) and non-virulent Bacille Calmette-Guerin (BCG) vaccine (ATCC 35734) were maintained in 7H9 medium with glycerol and OADC (oleic acid, albumin, dextrose, and catalase, BD Diagnostic Systems, New York, USA), subsequently the bacteria were harvested until the OD_600_ reached 0.6. Eight RMs were divided into three groups: phosphate buffer solution (PBS)-group, BCG-immunized group, and BCG mixed with BCG “adjuvant” BM2-immunized group^14^. Prior to vaccination, BCG was incubated with 5 μM of BM2 “adjuvant” in 100 μl of PBS at 37 °C for 1 h. At week -5, the RMs were subcutaneously vaccinated in the abdomen with BCG (10^6^ CFU BCG /100 μl/monkey) or BM2 + BCG (0.5 μM BM2 + 10^6^ CFU BCG /100 μl/monkey). Prior to the infection with *M. tb* H37Rv, IFN-γ production in the peripheral-blood-mononuclear cells (PBMCs) was detected using ELISA (Mabtech, Sweden) in these three groups^29^, and the results showed that BCG, BCG + BM2 groups were successfully immunized, compared to the PBS group^14^.

According to our previous studies^14,30^, at week 0, all RMs were intratracheally infected with 100 CFUs of *M. tb* H37Rv to deep lung using a fiber bronchoscope to generate an acute pulmonary tuberculosis model. At week 6, the PPD skin test demonstrated that all RMs were successfully infected with virulent *M*. *tb* H37Rv. Moreover, the body weight, erythrocyte sedimentation rate (ESR), and chest CT obtained using an Animage FIDEX Cone Beam CT scanner (designed for veterinary use, Animage, LLC, Pleasanton, CA, USA) also certified successful infection^14,28^. An animal laboratory technician obtained 5-ml of peripheral blood prior to infection (0 weeks) and post infection (3, 5, 10, 16, and 19 weeks) from the left femoral vein, and the blood samples were maintained in anticoagulative tubes containing EDTA-2K. These banked-blood samples were used for qPCR and ddPCR assays in the present study. The bacterial CFU burden in the lung, IFN-γ production by monkey-PBMC, PPD skin test, computed tomography (CT), and histopathological analysis of lung tissues have been shown to confirm BCG/BCG + BM2 groups elicited protective efficiency, compared with the PBS group^14^. All animal experiments were conducted in an Animal Biosafety Level 3 Laboratory (ABSL-III) of the Wuhan University School of Medicine.

A total of 200 μl whole blood was extracted using the DNeasy Blood and Tissue Kits (Qiagen, Germany) for each animal blood or tissue-DNA extraction. The cyclic system and amplification conditions for qPCR and ddPCR were the same as described above.

### Optimization of the PCR reaction system

The primers and probes were synthesized at Invitrogen (Shanghai, China). Full-length CFP10 (Rv3874), Rv1768, and EAST6 genes were amplified from a virulent *M. tb* H37Rv strain (ABSL III laboratory of Wuhan University) and cloned into the prokaryotic expression vector, pET28a, using DNA ligase (TSINGKE, Wuhan, China) to construct recombinant plasmids pET28a-CFP10, pET28a-Rv1768 and pET28a-EAST6. The recombinant plasmid-DNA was extracted using a plasmid extraction kit (Axygen, New York, USA) and confirmed by sequencing. The recombinant plasmids were used as templates in the PCR reaction and their concentrations were measured using the NanoDrop 2000 Ultramicro spectrophotometer. Different concentrations of probes (100, 150, 200, 250, 300, 350, 400, and 450 nmol/L) were used to determine the optimal amplification. Additionally, the optimum annealing temperature (55–65 °C) of different primers was determined using gradient temperature PCR.

### Real-time fluorescence quantitative PCR (qPCR)

Virulent *M. tb*-specific CFP10, Rv1768 and EAST6-DNA copy numbers were quantified using the Step One Plus real-time thermal cycler (ABI, Waltham, MA, USA). The primers and probes of Rv1768 are as follows: S: 5’-CGGCAACAGATTTGGCGAA

CA-3′, A: 5′-CGCTCCGAACAACGCGGCTAT-3′, probe: 5′- TTAGTGCAGCCAACGCGGCCGCG; the primers and probes of CFP10 are as follows: S: 5′- AAGCAGCCAATAAGCAGAAGC-3′, A: 5′- AGCCCATTTGCGAGGACA-3′, probe: 5′-GACGAATATTCGTCAGGCCGG-3′; and the primers and probes of EAST6 are as follows: S: 5′-CGTCCATTCATTCCCTCCTT-3′, A: 5′-TACGCCTCCGAACCGCTA-3′, Probe :5′-AAGCAGTCCCTGACCAAGCTCGCA-3′. The probes were marked with FAM at 5′ terminal and BQ1 at 3′ terminal. The amplification reaction included the following components: 10 μl of 2 × TaqMan Gene Expression Master Mix (Invitrogen, Shanghai, China), 2 μl of 100 ng/μl whole-blood genomic DNA or plasmid DNA, 1 μl of 10 μM forward primer and reverse primer, 0.5 μl of 10 μM TaqMan probe, and ddH_2_O to a final volume of 20 μl. The following thermocycler parameters were used: 95 °C for 5 min, followed by 40 cycles at 95 °C for 15 s, and 60 °C for 1 min. The standard curves for CFP10, Rv1768 and EAST6 genes were generated using known concentrations of the pET28a-CFP10, pET28a-Rv1768, and pET28a-EAST6 plasmids, and the CT values (Step One Plus build-in software) of the blood samples were transformed to the target gene copy numbers, based on the standard curves. Each sample was analyzed in three repetitive wells in one experiment, and each experiment was repeated three times.

### Droplet digital PCR (ddPCR)

Next, CFP10 and Rv1768 DNA copy numbers were quantified in the same samples using the QX100™ Droplet Digital™ PCR system (Bio-Rad, CA, USA). The 20 μl ddPCR mixture comprised of 10 μl of 2 × ddPCR Supermix (Bio-Rad, CA, USA), 1.0 μl of 10 μM sense and antisense primer, 0.5 μl of 10 μM probe, 2 μl of 100 ng/μl whole-blood genomic DNA or plasmid DNA, and RNase/DNase-free water to a final volume of 20 μl. The mixture was placed into the DG8 cartridge with 70 μl of droplet generation oil (Bio-Rad, CA, USA), and the droplets were formed in the droplet generator (Bio-Rad, CA, USA). Subsequently, the droplets were transferred to a 96-well PCR plate (Eppendorf, Hamburg, Germany), and PCR amplification was performed on a S1000 thermalcycler (Bio-Rad, CA, USA) using the following parameters: 95 °C for 5 min, followed by 40 cycles at 95 °C for 30 s and 60 °C for 60 s, with a final hold for 10 min at 98 °C. After amplification, the plate was loaded on the droplet reader (Bio-Rad, CA, USA), and the droplets from each well of the plate were automatically read at a rate of 32-wells per hour. The ddPCR data were analyzed using QuantaSoft analysis software (Bio-Rad, CA, USA), and the quantification of the target genes is presented as the number of copies per μl in PCR mixture. The recombinant plasmids were used as templates to draw the standard curves.

### Statistical methods

SPSS17.0 statistical analysis software was used to analyze the data. *T* test and ANOVA were used to compare the differences between each group. Differences were considered statistically significant for *p* values <0.05.
